# Anti-NMDAR encephalitis with simultaneous hypertrophic pachymeningitis in a 68-year-old male: a rare case report

**DOI:** 10.1186/s12883-019-1444-x

**Published:** 2019-08-31

**Authors:** Huafang Jia, Xiaoli Xie, Faying Qi, Long Wang, Lijuan Wang, Fengyuan Che

**Affiliations:** 1 Department of Neurology, The Eleventh Clinical Medical College of Qingdao University, Linyi People’s Hospital, 27 East Section of Jiefang Road Lanshan District, Linyi, 276000 Shandong China; 2 Central Laboratory, Linyi Key Laboratory of Neurophysiology, Linyi People’s Hospital, 27 East Section of Jiefang Road Lanshan District, Linyi, 276000 Shandong China

**Keywords:** Anti-NMDA receptor encephalitis, Autoimmune encephalitis, Hypertrophic pachymeningitis (HP), Dural hypertrophy, NMDAR

## Abstract

**Background:**

Anti-N-methyl-D-aspartate receptor (NMDAR) encephalitis is one of the most frequent types of autoimmune encephalitis. However, the instigating mechanisms are as yet not fully ascertained. Cardinal clinical manifestations of anti-NMDAR encephalitis include acute behavioural change, psychosis, and catatonia. As the level of diagnosis increases, encephalitis becomes more common, but there are never been published in patients with anti-NMDAR encephalitis and simultaneous hypertrophic pachymeningitis.

**Case presentation:**

A sixty-eight-year-old man who presented with mental, behavioral abnormalities, unstable walking, headaches, and erratic hand movements. The neuropsychiatric symptoms and cerebrospinal fluid examination was consistent with the diagnosis criteria of anti-NMDAR encephalitis. Magnetic resonance imaging of the brain showed a thickening of dura mater localized at the left tentorium cerebelli, left cerebral hemisphere, and cerebral falx; the thickening dura mater was characterized by an intense contrast enhancement after the administration of gadolinium. High doses of intravenous methylprednisolone were administrated during his hospitalization. After 5 days, the patient’s condition improved.

**Conclusions:**

We herein describe a rare case of a 68-year-old man with anti-NMDAR encephalitis presenting with concomitant hypertrophic pachymeningitis. We systematically expounded anti-NMDAR encephalitis and hypertrophic pachymeningitis, and made bold conjectures on the etiology and pathogenesis of these two diseases, hoping to stimulate new ideas from clinicians and basic medical researchers.

## Background

Anti-N-methyl-D-aspartate receptor (NMDAR) encephalitis is an autoimmune nervous system disease mediated by NMDAR antibodies. It is one of the most frequent types of autoimmune encephalitis. Patients often present with subacute psychiatric symptoms, memory loss, movement disorders, and seizures [1]. Since its recognition, clinicians have inreasingly identified the disease and there has been a rapid surge in the number of cases reported. Hypertrophic pachymeningitis (HP) is a relatively rare condition that causes localized or diffused thickening of the dura mate [2]. The dominant clinical symptoms are chronic headaches with or without neurological manifestations [3].

Several cases of anti-NMDAR encephalitis and those of HP have previously been published. In fact, to our knowledge, simultaneous occurrence of both conditions has never been published before. We herein describe the case of a 68-year-old man with anti-NMDAR encephalitis presenting with concomitant HP. We discuss the causes, presentation, and treatment in patients with anti-NMDAR encephalitis and HP.

## Case presentation

A 68-year-old man was admitted to the Department of Neurology, Linyi People’s Hospital in 15 May of 2018 with mental and behavioral abnormalities, unstable walking, headaches, and erratic hand movements. Family members complained that over the past 2 months, the patient had been showing impulsiveness and irritability, wastefulness, particularly with food, and using foul language. These behaviours commonly lasted a half an hour each time, and the patient would often be conscious of his actions afterwards. He reported that he frequently suffered from mild headaches in the left frontal occipital region, numbness in the left facial face, and urinary incontinence at night. He had no known allergies. He was raised and lived in the area. He has been smoking for 30 years but but did not drink alcohol or use any illicit drugs. He had not traveled recently, and reported no exposures to patient with similar symptoms, farm or livestock, or bitter insect bites. There was no family history of genetic diseases and autoimmune diseases.

On examination, his temperature was 37 °C, blood pressure was 90/53 mmHg, heart pulse was 99 beats per minute, respiratory rate was 21 breaths per minute. The patient was fully awake and communicated with the doctor in a normal way, but was slow to respond, had poor memory and computational power. He had a stiff face, nuchal rigidity, and mild ptosis on the left side. The bilateral pupils were normal. Sensory examination revealed hypoesthesia on the left side of the face. The strength of the limbs was normal. No pathological reflexes were detected. Cerebellar testing was not carried out because the patient was not cooperative.

Routine blood, No abnormalities in liver, kidney and thyroid function tests. The levels of albumin, globulin, electrolytes and glucose in the blood are also normal. Plasma C-reactive protein (CRP) was 98 mg/l, Erythrocyte sedimentation rate (ESR) was 30 mm/h. Autoimmunity exams revealed that anti-NMDAR antibodies were positive and levels of serum IgG4 was 60.5 mg/dL. whereas serum values were negative for tumor markers (AFP, CEA, CA125, CA199, FPSA, NSE, CYFRA21-1, CA72–4), rheumatoid factor (RF), antineutrophil cytoplasmic antibodies (ANCA), antinuclear antibodies (ANA), MPO antibodies and PR3 antibodies.

**Fig. 1:**
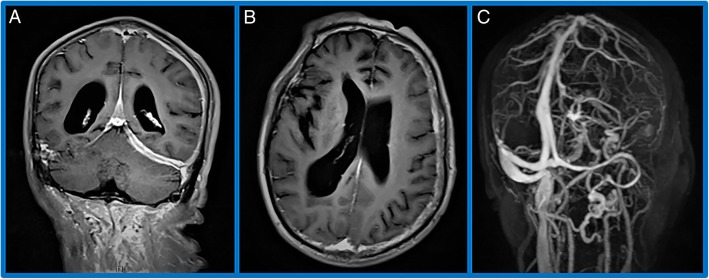
Magnetic resonance imaging of the brain: a thickening of dura mater localized at the left tentorium cerebelli, left cerebral hemisphere, and cerebral falx; the thickening dura mater was characterized by an intense contrast enhancement after the administration of gadolinium (**a, b**). Magnetic resonance venography: no obvious indication of the left internal jugular vein, and both of the left transverse sinus and the sigmoid sinus was slim with local stenosis; there was also increased cortical drainage in the left cerebral hemisphere (**c**)

Lumbar puncture and subsequent cerebrospinal fluid (CSF) examination results showed a raised protein level (831 mg/L), white blood cell count was 8000 with mainly lymphocytes, and no abnormal performance from infection. An immunological examination of the CFS results also tested positive for anti-NMDAR antibodies, whereas IgG4 and anti-CASPR2, anti-AMPA1, anti-AMPA2, anti-LGI1, anti-GABAB antibodies were normal. There was no lesion suggestive of lung disease in Chest High Resolution CT. Magnetic resonance imaging (MRI) of the brain showed a thickening of dura mater localized at the left tentorium cerebelli, left cerebral hemisphere, and cerebral falx; the thickening dura mater was characterized by an intense contrast enhancement after the administration of gadolinium (Fig. 1a, b). Magnetic resonance venography (MRV) showed no obvious indication of the left internal jugular vein, and both of the left transverse sinus and the sigmoid sinus was slim with local stenosis; there was also increased cortical drainage in the left cerebral hemisphere (Fig. 1c). Video electroencephalogram (EEG) monitoring showed that each electrode detected a small amount of low-amplitude slow wave.

The patient underwent a composite evaluation and a diagnosis of anti-NMDAR encephalitis with dural hypertrophy was proposed. Following recent research findings and expert advice, the patient received high doses of intravenous methylprednisolone during his hospitalization. No adverse or unexpected events occurred during the treatment. After 5 days, the patient’s condition improved and he was asked to leave the hospital. The results of the MRI resonance examination showed almost the same as before the treatment. The patient is very satisfied with the treatment and treatment results received. After hospital discharge, 30 mg/die of prednisone, per os was recommended. The patient was referred to us again at 10 months after discharge. There had been no symptoms include mental and behavioral abnormalities. Nevertheless, slight chronic headache persisted. A follow-up MRI did not find Significant changes. Prednisone was followed by maintenance treatment. Follow-up information through telephone call showd partial regression of headaches in 1 August of 2019.

## Discussion and conclusions

We report the case with anti-N-methyl-D-aspartate receptor (NMDAR) encephalitis and simultaneous HP. Our patient who presented with episodic abnormal mental behavior with unstable walking, the CSF anti-NMDAR antibodies is positive. Magnetic resonance imaging (MRI) of the brain showed a thickening of dura mater localized at the left tentorium cerebelli, left cerebral hemisphere, and cerebral falx; the thickening dura mater was characterized by an intense contrast enhancement after the administration of gadolinium. Dural hypertrophy is a clinical manifestation of HP. In actuality, HP is a potential manifestation of numerous clinico-pathological entities including infectious diseases, autoimmune disorders, malignant neoplasms, and no obvious cause of idiopathic hypertrophic pachymeningitis [4]. The pathogenic mechanism of HP is obscure, but more and more evidence suggests that abnormal autoimmunity may play an important role [5]. Hypertrophic pachymeningitis has been reported to be associated with a variety of autoimmune disease, including rheumatoid arthritis, sarcoidosis, IgG4-related disorder, Sjögren’s syndrome, Rosai-Dorfman disease, antiphospholipid syndrome and anti-centromere antibody-positive status [3, 6–10]. In our patient, the serum and CSF IgG4 level were within the normal range. Serum values were negative for RF, ANCA, ANA, MPO antibodies and PR3 antibodies. We are very regret that the patient did not have a histological proof of HP because of various restrictions and his personal wishes, so we can only diagnose HP from the imaging level. According to Dalmau J et al. MRI showed that 14 of the 100 patients had an enhanced contrast of the overlaying meninges [11]. Irani et al. speculated that the dura mater lacking the blood-brain barrier may be one of the first sites of inflammation in anti-NMDAR encephalitis through clinical observation of disease progression [12]. Suzuki et al. reported a case of anti-NMDAR encephalitis preceded by dura mater lesions and predicted that the dura mater lesions on MRI may be available diagnosis and give insight into the the etiology and pathogenesis of anti-NMDAR encephalitis [13]. In our experience, it is unclear whether dural hypertrophy was a manifestation of anti-NMDAR encephalitis or merely coincidental. Unexpected discovery, the performance of MRV does not rule out venous thrombosis due to HP, which shows no development of the left internal jugular vein and local stenosis of the left transverse sinus and sigmoid sinus and increased cortical drainage of the left cerebral hemisphere. While the overlap may have been occasional, one could raise the consideration of a congenerous pathogenic mechanism, owing to an underlying disturbances of immune mechanisms. Our next step is to conduct an in-depth study of the relevance of these two diseases from multiple perspectives.

## Data Availability

All data generated or analysed during this study are included in this published article.

## Abbreviations

CSF: cerebrospinal fluid
EEG: electroencephalogram
FLAIR: fluid-attenuated inversion recovery
HP: Hypertrophic pachymeningitis
MRA: Magnetic resonance angiography
MRI: Magnetic resonance imaging
MRV: Magnetic resonance venography
NMDAR: N-methyl-D-aspartate receptor

## References

[1] Titulaer MJ, McCracken L, Gabilondo I, Armangue T, Glaser C, Iizuka T (2013). Treatment and prognostic factors for long-term outcome in patients with anti-NMDA receptor encephalitis: an observational cohort study. Lancet Neurol.

[2] D'Andrea G, Trillo G, Celli P, Roperto R, Crispo F, Ferrante L (2004). Idiopathic intracranial hypertrophic pachymeningitis: two case reports and review of the literature. Neurosurg Rev.

[3] Kupersmith MJ, Martin V, Heller G, Shah A, Mitnick HJ (2004). Idiopathic hypertrophic pachymeningitis. Neurology..

[4] De Virgilio A, de Vincentiis M, Inghilleri M, Fabrini G, Conte M, Gallo A (2017). Idiopathic hypertrophic pachymeningitis: an autoimmune IgG4-related disease. Immunol Res.

[5] Dumont AS, Clark AW, Sevick RJ, Myles ST (2000). Idiopathic hypertrophic pachymeningitis: a report of two patients and review of the literature. Can J Neurol Sci.

[6] Li JY, Lai PH, Lam HC, Lu LY, Cheng HH, Lee JK (1999). Hypertrophic cranial pachymeningitis and lymphocytic hypophysitis in Sjogren’s syndrome. Neurology..

[7] Bruggemann N, Gottschalk S, Holl-Ulrich K, Stewen J, Heide W, Seidel G (2010). Cranial pachymeningitis: a rare neurological syndrome with heterogeneous aetiology. J Neurol Neurosurg Psychiatry.

[8] Tokushige S, Matsumoto H, Takemura T, Igeta Y, Hashida H (2012). Secondary hypertrophic pachymeningitis in antiphospholipid syndrome. J Neuroimmunol.

[9] Breiner A, Dubinski W, Gray B, Munoz DG (2013). A 63 year old woman with white matter lesions and pachymeningeal inflammation. Brain Pathol.

[10] Yokoseki A, Saji E, Arakawa M, Kosaka T, Hokari M, Toyoshima Y (2014). Hypertrophic pachymeningitis: significance of myeloperoxidase anti-neutrophil cytoplasmic antibody. Brain J Neurol.

[11] Dalmau J, Gleichman AJ, Hughes EG, Rossi JE, Peng X, Lai M (2008). Anti-NMDA-receptor encephalitis: case series and analysis of the effects of antibodies. Lancet Neurol.

[12] Irani SR, Bera K, Waters P, Zuliani L, Maxwell S, Zandi MS (2010). N-methyl-D-aspartate antibody encephalitis: temporal progression of clinical and paraclinical observations in a predominantly non-paraneoplastic disorder of both sexes. Brain J Neurol.

[13] Suzuki H, Kitada M, Ueno S, Tanaka K, Kusunoki S (2013). Anti-NMDAR encephalitis preceded by dura mater lesions. Neurol Sci.

